# Mental health and trauma in asylum seekers landing in Sicily in 2015: a descriptive study of neglected invisible wounds

**DOI:** 10.1186/s13031-017-0103-3

**Published:** 2017-01-13

**Authors:** Anna Crepet, Francesco Rita, Anthony Reid, Wilma Van den Boogaard, Pina Deiana, Gaia Quaranta, Aurelia Barbieri, Francesco Bongiorno, Stefano Di Carlo

**Affiliations:** 1 Medici Senza Frontiere Missione Italia, via Cavour 147/6, 00185 Rome, Italy; 2 Médecins Sans Frontières Bruxelles, Operational Research Unit Luxembourg, 68 rue de Gasperich L-1617, Luxembourg City, Luxembourg; 3 Assessorato alla Salute Regione Sicilia (Local Ministry of Health), Piazza Ottavio Ziino 24, Palermo, 90145 Italy

**Keywords:** Immigration, Asylum seekers, Refugee, Mental health, Italy, Europe, Traumatic event, Operational research

## Abstract

**Background:**

In 2015, Italy was the second most common point of entry for asylum seekers into Europe after Greece. The vast majority embarked from war-torn Libya; 80,000 people claimed asylum that year. Their medical conditions were assessed on arrival but their mental health needs were not addressed in any way, despite the likelihood of serious trauma before and during migration. Médecins sans Frontières (MSF), in agreement with the Italian Ministry of Health, provided mental health (MH) assessment and care for recently-landed asylum seekers in Sicily. This study documents mental health conditions, potentially traumatic events and post-migratory living difficulties experienced by asylum seekers in the MSF programme in 2014–15.

**Methods:**

All asylum seekers transiting the 15 MSF-supported centres were invited to a psycho-educational session. A team of psychologists and cultural mediators then provided assessment and care for those identified with MH conditions. Potentially traumatic events experienced before and during the journey, as well as post-migratory living difficulties, were recorded. All those diagnosed with MH conditions from October 2014 to December 2015 were included in the study.

**Results:**

Among 385 individuals who presented themselves for a MH screening during the study period, 193 (50%) were identified and diagnosed with MH conditions. Most were young, West African males who had left their home-countries more than a year prior to arrival. The most common MH conditions were post traumatic stress disorder (31%) and depression (20%). Potentially traumatic events were experienced frequently in the home country (60%) and during migration (89%). Being in a combat situation or at risk of death, having witnessed violence or death and having been in detention were the main traumas. Lack of activities, worries about home, loneliness and fear of being sent home were the main difficulties at the AS centres.

**Conclusion:**

MH conditions, potentially traumatic events and post-migratory living difficulties are commonly experienced by recently-arrived ASs, this study suggests that mental health and psychosocial support and improved living circumstances should be integrated into European medical and social services provided by authorities in order to fulfil their humanitarian responsibility and reduce the burden of assimilation on receiving countries.

## Background

According to the United Nation High Commissioner for the Refugees (UNHCR) in 2015, 65.3 million individuals were forcibly displaced worldwide as a result of persecution, conflict, generalized violence, or human rights violations; this constitutes a steady increase from previous years [1]. Italy, after Greece, was in 2015 the main point of entry into Europe for people who are fleeing from war and economic instability. The main migratory route to Italy is across the Mediterranean Sea on overcrowded boats and small rubber dinghies from war-torn Libya and other North African countries.

Nationalities of migrants entering Europe have varied significantly over time, depending on the areas of conflict, political instability, human trafficking and changing migratory routes. A common characteristic of these migrants is that they are psychologically vulnerable due to trauma, torture, stay in detention or refugee camps with poor living conditions prior to arrival in Europe. In addition to that, migrants might feel stressed by social isolation, worries about family back home, and the complicated, lengthy asylum processes in the receiving countries [2–4].

A vast majority of migrants landing in Italy decide to stay and formally request asylum in Italy itself, thereby entering into the Italian immigration system. Asylum seekers (ASs) are defined as, “individuals who have sought international protection and whose claims for refugee status have not yet been determined” [1].

The Italian immigration reception system is rather fragmented since the government has outsourced the service delivery to private organizations [5]. This has created, notably in Sicily, a wide-spread business offering services to ASs with limited control from the Italian authorities. Although in theory health care provided by the national health services (SSN) is free of charge for ASs, access frequently does not exist in practice for various reasons [6]. This is especially true for mental health (MH) care services where language barriers and limited transcultural expertise play major roles.

In 2015, 99,096 ASs were hosted in the Italian AS centres, of whom 19% were located in Sicily [7]. Ragusa province in Sicily hosts about 600 people in 15 AS centers, *Centri di accoglienza straordinaria*, but accurate data was never provided by the Ministry of Health.

The vast majority of ASs hosted in Ragusa province landed in Pozzallo harbour and include men, women, children, unaccompanied minors and older people. In 2015 out of 55 landings, 49 were from Libya (Médecins Sans Fontières programme data). Libya has lately been a place where sub-Saharan Africans are persecuted and victims of violence and abuse, as reported by ASs themselves and documented in a report by the North Africa Mixed Migration Task Force [8].

For the last two decades MSF has been providing medical care in the first reception centres at the landing harbours and our doctors and nurses together with cultural mediators have seen a wide variety of the landing migrant population. [9] Scabies, burns and wounds were the most common physical conditions seen and dealt with. These are stigmata of detention, perilous journey in the sun-scalded rubber dinghies and across the desert. In several cases, unwanted pregnancies, multiple limb fractures and bullets lodged under the skin bore witness of violence experienced in Libya and long the journey (MSF programme data).

From July 2014 until February 2016, following an official agreement with the local Ministry of Health, the medical humanitarian organization, Médecins Sans Fontières (MSF), provided psychological support to ASs hosted at these 15 AS centers.

There is a vast literature written in the last twenty years concerning migrants and mental health conditions; many studies focus on the prevalence rate of MH conditions and risk factors related to them, Post Traumatic Stress Disorder (PTSD) and depression being the most represented and studied disorders [10–13].

According to different studies the prevalence rates can vary from 0–99% and 3–86% for PTSD and depression respectively and this is due to substantial intersurvey heterogeneity [11, 14]. In fact in two comprehensive systematic reviews, one by Steel [10] and one by Fazel [11], the variability of prevalence rates depends on methodological factors such as sample size, sampling methods and types of potentially traumatic events as well as substantive factors such as time since the conflict and country of origin. Prevalence rate will depend on the population exposure to the risk factors identified. In the same reviews, the most methodologically robust surveys find a 15–20% median prevalence of PTSD and depression [15].

There are also many other MH conditions such as unexplained somatic disorders, anxiety, psychosis, illicit drug and harmful alcohol use, that are more frequent in migrants than in the general population. However, for drug and alcohol misuse the evidence based is weak and further quality research needs to be conducted [16, 17].

Higher exposure to potentially traumatic experiences and post-migration stress are the most common factors associated with higher rates of MH disorders across several studies [4, 18]. In a systematic literature review by Hassan looking at mental health and psychosocial wellbeing of Syrians affected by armed conflict [19], MH problems can be broadly categorised in three: exacerbation of pre-existing MH disorders, new problems caused by conflict, displacement and multiple losses and finally issues related to adaptation to post-emergency context.

There are also substantial differences between refugees and asylum seekers as the former have obtained a formal recongition of international protection (refugee status) whereas the latter don’t have their status recognised yet and they might never do so. ASs carry a more recent history of potentially traumatic experiences and have not taken part into a social net in the receiving country, thus, face a greater uncertainly about their future.

A study comparing these two groups in a population of Afghans, Iraqi and Somalians living in the Nederlands described that more ASs compared to refugees reported poor general health status, and more symptoms of PTSD, depression and anxiety [20].

Most studies on asylum seekers were conducted in northern European countries and concerned Middle Eastern nationals, whereas little has been written in Italy where many Sub-Saharan Africans tend to claim asylum [8, 10]. However, one Italian study by Aragona et al. described the impact of post migration living difficulties on somatization in 101 migrants accessing a primary care service [11].

In addition, almost all papers reported study populations drawn from “settled” migrants, rather than those recently-arrived in Europe [9, 12, 13]. It is likely that the MH needs of recently-arrived ASs would be different from those of a settled population, and it is important to document the burden of psychological issues if receiving countries are to cope successfully with a large influx of people with potentially traumatic stories.

Therefore, the aim of this study was to describe the MH conditions detected and traumas reported in a population of ASs shortly after landing in 15 MSF-supported centers in Sicily, Italy. Specific objectives were to describe the frequency and types of, 1. MH conditions, 2. potentially traumatic events suffered by the ASs before leaving their country of origin and during their migration journey and 3. post-migratory living difficulties experienced in Italy.

## Methods

### Study design

This was a descriptive, cross-sectional study using routinely-collected programme data.

### Setting

#### General

Migrants who claim asylum in Italy are hosted in facilities run by government-selected private companies that should provide housing, food, Italian language classes, presence of cultural mediators, socio-legal needs and access to health care. Asylum seekers remain in these facilities until the process to obtain a form of humanitarian or international protection is completed. As this process is very bureaucratic, it might take from six months to over a year or even longer, especially when asylum is denied and a reappeal is undertaken. Health care in Italy is free of charge and delivered by the National Health Service (SSN) and is theoretically available to all ASs.

#### Specific

In Ragusa province the AS centres are usually located in remote countryside areas. While housing and food are regularly provided, Italian language classes, socio-legal assistance and access to health care are very limited or completely lacking. This results in dependency and uncertainty for the AS. There is a particular lack of access to psychological care, as no psychologists, counsellors or (to less extent) cultural mediators are employed in the AS centres despite it being part of the centre’s charter.

#### MSF psychological assessment and care

To address MH needs within the AS centres in Ragusa province, the psychologists gave a training session to the general staff working in the AS centres on the impact of forced migration on psychology of asylum seekers and how to recognise MH symptoms.

**Fig. 1:**
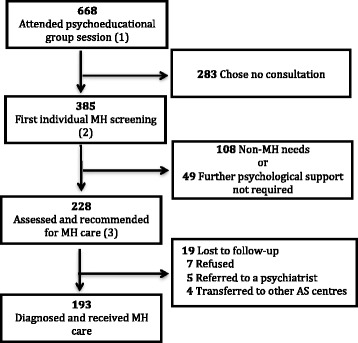
Mental Health patient flow, asylum seekers in 15 centres Ragusa Italy (October 2014, December 2015). MH: Mental Health, AS Asylum Seekers. The intervention consisted of psycho-educational group sessions (step1), followed by first individual MH screening (step2) and short cycle of psychological consultations (step3)

As part of the MSF MH service, the psychologists offered a tiered approach to providing psychological care. It began with psycho-educational group sessions (step 1 of Fig. 1) run by two clinical psychologists together with cultural mediators at all the AS centres. Topics discussed were MSF’s humanitarian role, basics on the asylum process in Italy, effects of potentially traumatic events on mental health, the role of psychologists and how to access MH support for the ASs. The group sessions aimed to identify ASs psychologically vulnerable and strengthen their coping mechanisms and resilience. They were offered every time there were new arrivals at the AS centres.

People who attended the psycho-educational groups could self-refer for a first individual MH screening by a psychologist (step 2 of Fig. 1) if they wished; in addition, people who were identified by AS centre staff or the psychologists as needing MH support were invited to attend the first individual MH consultation.

The psychologists used semi-structured interview to screen ASs for significant MH symptoms and those who were felt to need a MH consultation were advised to attend a follow-up MH consultation for diagnosis and treatment (step 3 of Fig. 1). At the same time, the psychologists provided self-help tools for those who had MH concerns but didn’t need a MH follow-up. An AS could also request a repeat psychological assessment at a later stage. At the completion of their therapy sessions, patients were given a clinical report, which could be presented to the asylum commission.

As part of the MSF MH programme, informative sessions on various subjects concerning mental health and ASs were offered to all the personnel working in the AS centres and local immigration authorities. Particular focus was on the effects of potentially traumatic events on human psychology and how to recognise the need for psychological support.

The two psychologists, both speaking English and French, with experience in ethnopsychiatry worked in parallel and related to the ASs taking into account the person’s cultural beliefs. Their ethnopsychiatric perspective allowed the psychologists to deconstruct their Western nosographic categories in order to interact with the cultural categories of the patient in a sort of translation.

A pool of cultural mediators was employed in order to translate the main languages of the ASs: Mandinga, Bambarà, Poular, Soninke, Wolof, Bangla, Urdu, Farsi, Arabic, Tigrigna and Somalian.

A cultural mediator is a person that shares the same geo-cultural origins with the AS and makes the communication between the health worker and the migrant possible, not just as a simple linguistic interpreter but also as a cultural facilitator. He/she acts as a bridge in between two worlds thanks to the knowledge and experience of them both.

They had all been asylum seekers themselves a few years beforehand and had been officially recognized as refugees in Italy. Hardly any of them had previously received formal training in cultural mediation but MSF pioneered a few training sessions given by a formally-trained, experienced cultural mediator. On one hand the mediators were trained and coached by the psychologists to interpret psycho-educational messages at the group sessions and they mediated in the MH consultations, while on the other hand the psychologists were coached by the cultural mediators in cultural sensitivity. Both became an inseparable assessment and treatment pair.

In order to determine MH conditions and the need for further consultations, the psychologists based their diagnostic assessment on the Diagnostic and Statistical Manual of Mental Disorders-5 [1] (DSM-5) criteria. There was cross-checking between the two psychologists regarding diagnosis only for more complex cases, given workload limitations.

A self-reported questionnaire (SRQ-20 designed by the World Health Organisation) was initially used for the individual screening [21], but was found to be unsuitable for the context of recently-arrived ASs from diverse ethnic and cultural backgrounds, hence it was soon abandoned.

The MSF psychologists worked in synergy with the psychiatrist of *Medici per i Diritti Umani*, an Italian NGO present in the same geographical area. Likewise, for socio-legal issues and informative sessions concerning adults, children and vulnerables, we combined our efforts respectively with UNHCR, Save the Children and IOM, in fact all three actors monitored arrivals at the landing harbour and, to less extent, the ASs’ centres.

### Study population

All patients diagnosed with MH conditions and followed up by the psychologists from October 2014 to December 2015 were included in the study.

### Measures

For all MH patients general sociodemographic characteristics plus language used for the MH consultation and whether or not there was involvement of a cultural mediator were included.

The duration of the migration journey was self-reported. Time spent residing in transit countries, even for work, was considered part of their migration journey. Duration of stay in Italy was calculated from the date of arrival in Italy documented on the immigration papers. AS’ vulnerability status (pregnant/unaccompanied minor/disable) was identified following UNHCR definitions.

For the main and secondary MH conditions, the psychologists referred to the DSM-5 Manual, except for ethnopsychiatric conditions and psychological distress in the absence of a diagnosable mental disorder.

Up to two concurrent diagnosis were recorded. The number of MH consultations required by each patient, psychopharmacological treatments and referral to a psychiatrist were also documented.

Outcomes were defined according to the psychologists’ clinical judgement (improved, recovered, unchanged condition) and information given by the staff at the AS centre (transferred to other AS centres, escaped).

Up to three potential traumatic events per person, before leaving the country of origin and during the migration journey to Italy, were self-reported. An event was defined as potentially traumatic by the psychologists if it resulted in a serious disruption, a fracture in the individual’s life. The individual traumatic events were then grouped into wider categories according to the Harvard Trauma Questionnaire [22]. The definition of torture employed was: ‘Severe pain or suffering, whether physical or mental, inflicted for such purposes as obtaining information or a confession, exerting pressure, intimidation or humiliation’ [23].

Similarly, up to three post-migratory living difficulties (PMLD) in the AS centres were recorded based on the difficulties reported by the patients and on the psychologists’ clinical impressions. A PMLD was defined as a severe stressor in a patient’s life.

### Data sources and handling

Patients’ data were recorded by the MSF psychologists during each consultation and entered manually onto paper registers. They were then single-entered into a dedicated Excel database (Microsoft Excel, 2011) on a weekly basis. The same data source was used for initial assessment and follow up visits. Dates of arrival in Italy were obtained from the immigration police.

### Statistical methods

The dataset was analysed in Excel (Microsoft Excel, 2011). Categorical variables were summarized using frequencies and proportions; medians and interquartile ranges were reported for skewed continuous variables. Sample size calculation was not required as all patients were included.

## Results

AS patient flow in the MSF MH programme is shown in the Fig. 1. A total of 668 patients were exposed to the psycho-educational session led by the psychologists. From those sessions 385 presented themselves for a first MH screening visit, and the psychologists identified 232 (60% of total screened) patients with mental health symptoms for whom follow-up assessment and consultation was recommended. Finally, 193 (50% of total screened) patients were taken into care and diagnosed with mental health conditions, while the rest were either lost to follow-up, refused treatment, were referred directly to a psychiatrist or transferred to other centres.

Socio-demographic characteristics of all 385 asylum seekers screened for mental health conditions and 193 patients in care are shown in Table 1. Of the 193 in care, the majority were males (92%) had a median age of 23, and were West Africans (83%) with Nigeria, Gambia and Senegal being the most common countries of origin. In terms of civil status, 74% were single and the rest had left spouses in the country of origin. ASs with a vulnerability accounted for 12%. The duration of the journey from the country of origin to Italy was more than 12 months in a large proportion (57%). The median length of stay in Italy up to the time of MH assessment was 74 days. Cultural mediators were used during the individual MH consultations for 30% of ASs either because they did not speak English or French or because transcultural facilitation was required.

**Table 1:** Sociodemographics, asylum seekers screened and diagnosed with mental health conditions Ragusa (October 2014 December 2015)

Characteristic	Screened for MH conditions	Diagnosed with MH conditions
	*N* = 385 (%)	*n* = 193 (%)
Gender		
Female	34 (9)	15 (8)
Male	351 (91)	178 (92)
Age in years (median; IQR)	23 (20–27)	23 (20–26)
Country of origin		
Nigeria	77 (20)	40 (21)
Gambia	65 (17)	38 (20)
Senegal	51 (13)	32 (17)
Mali	47 (21)	20 (10)
Bangladesh	43 (11)	11 (6)
Other West African^a^	58 (15)	30 (16)
Other^b^	41 (11)	21 (11)
Unknown	3 (1)	1 (1)
Vulnerability (disabled, pregnant, unaccompanied minors)	34 (9)	23 (12)
Length of stay in Italy in days (median; IQR)	77 (45–121)	74 (43–118)
Duration of journey from origin country to Italy		
0–6 months	102 (26)	48 (25)
6–12 months	70 (18)	33 (17)
> 12 months	208 (54)	110 (57)
Unknown	5 (1)	2 (1)

*IQR* interquartile range, *MH* mental health

^a^Ivory Coast, Guinea Conakry, Guinea Bissau, Liberia, Ghana

^b^Pakistan, Eritrea, Somalia, Egypt, Afghanistan, Morocco, Libya

As shown in Table 2, the most common diagnoses were PTSD (31%) and depression (20%) while two thirds of patients had at least two MH conditions. Severe depression was the diagnosis for 12% of cases and beside PTSD the other trauma- and stressor-related disorders (i.e., acute stress and adjustment disorders) were occasionally diagnosed. A referral to a psychiatrist was needed in 22% of cases and psychopharmacological treatment was used for 18%. The 36 people on treatment were taking the following medications: antidepressants (14), antipsychotics (4), anxiolytics (10) and mood stabilisers (8).

**Table 2:** Clinical characteristics of patients diagnosed with mental health conditions, Ragusa (October 2014 December 2015)

Characteristic	*N* = 193 (%)
MH main diagnosis^a^	
Post Traumatic Stress Disorder	59 (31)
Depression	38 (20)
Anxiety disorders	21 (11)
Psychological distress^c^	22 (11)
Somatoform disorder	15 (8)
Sleep-wake disorder	13 (7)
Other^d^	25 (13)
MH second^e^ diagnosis^a^	
Traumatic Stress Disorder and related disorders^b^	3 (2)
Depression^b^	42 (22)
Anxiety disorders	20 (10)
Somatoform disorder	10 (5)
Psychological distress	3 (2)
Sleep-wake disorder	27 (14)
Other^d^	24 (12)
None^f^	64 (33)
Two concurrent MH diagnosis	129 (67)
Referrals to psychiatrist	43 (22)
Psychopharmacological treatment	36 (18)
Number of MH consultations per person (median; IQR)	4 (3–6)

*MH* Mental Health, *IQR* interquartile range

^a^MH diagnosis according to the Diagnostic and Statistical Manual of Mental Disorders (DSM 5) except from psychological distress and ethnopsychiatric conditions (Other)

^b^Includes mild, moderate, severe

^c^Exclusion diagnosis

^d^Ethnopsychiatric conditions, personality, psychotic, cognitive, dissociative, acute stress, adjustment and substance-related disorders

^e^Concurrent diagnosis, secondary importance

^f^Patients with only a MH main diagnosis

The median number of MH consultations was four per person.

The outcomes for the 193 patients were: 130 (44%) people improved or recovered in their MH conditions, nine remained unchanged, 10 were referred to dedicated MH vulnerable centres, nine deliberately left the centres, seven went under exclusive care of a psychiatrist, four refused to continue MH care, 23 were transferred to other centres before completion of MH treatment and for one patient, the outcome was unknown.

Potentially traumatic events (PTEs) prior to departing the country of origin were experienced by a large percentage of ASs (60%), while PTEs that occurred during the migration journey were experienced by an even larger percentage (89%), in the majority of cases ASs experienced more than one event per person and very few did not experience any PTEs.

Table 3 shows the type of potentially traumatic events that occurred in the country of origin, the most common being in a combat situation or being at risk of death (23%), followed by having witnessed violence or death (15%). The most frequent event experienced during the journey was being in a combat situation or at risk of death (29%), followed by detention or kidnapping (24%). Torture was suffered by 11% of ASs at some point in their migration journey.

**Table 3:** Potentially traumatic events experienced by patients with mental health conditions, Ragusa (October 2014 December 2015)

Traumatic events	Before leaving the home country	During the migration journey
	*N* = 273^a^ (%)	*N* = 434^a^ (%)
Combat situation or at risk of death	64 (23)	127 (29)
Witnessed violence or death	42 (15)	59 (14)
Relative killed/missing/incarcerated	42 (15)	14 (3)
Intra-familial conflict	41 (15)	–
Physical/psychological violence	20 (7)	44 (10)
Rape or sexual abuse	–	10 (2)
Detention/kidnapping	12 (4)	102 (24)
Torture	4 (1)	47 (11)
Other situations that caused fear for life^b^	48 (18)	31 (7)

^a^N represents potentially traumatic events (PTEs) and not patients. The proportions do not add up to 100% as multiple PTEs (up to three) were possible for an individual

^b^Includes forced labour, witchcraft, persecution due to homosexuality, etc

**Table 4:** Post-migration life difficulties experienced by patients with mental health conditions, Ragusa (October 2014 December 2015)

Post-migration life difficulties	*N* = 193 (%)^a^
Difficulties with the asylum process:	
Fear of being sent home	35 (18)
Distress related to denial of asylum claim	27 (14)
Asylum commission took a long time	17 (9)
Power abuse/intimidation	6 (3)
Conflict with immigration authorities	4 (2)
Difficulties related to the AS centre:	
Lack of daily activities	51 (26)
Conflict with team of centre	29 (15)
Concerns about family:	
Worries about family back home	39 (20)
News of death of family and friends	7 (4)
Subjective stressors:	
Loneliness and boredom	35 (18)
Feeling neglected	33 (17)
Adjustment difficulties	31 (16)
Feeling you cannot control events in your life	27 (14)
Feeling that injustice is done to you	26 (13)

^a^these proportions do not add up to 100% as multiple PMLDs were possible for an individual

Life in the reception centres was fraught with difficulties, as reported by 89% (Table 4). The most common types were lack of daily activities (26%), worries about home (20%), loneliness and boredom (18%), and fear of being sent home (18%). Overall, 42% of ASs reported having fear for the future in general.

## Discussion

To our knowledge this is the first mental health study of recently-arrived ASs landing in Sicily during 2014–2015, and it reveals a high burden of mental health diagnoses amongst the selective group of ASs that was screened. It also documents high levels of potentially traumatic events experienced before and during the migration journey, and high stressors after migration. It is important as it implies that care of ASs should go beyond physical needs and anticipate psychological and mental health problems. It also may explain some behaviours of migrants leading to better understanding of their situation and improved care by receiving agencies and personnel.

The majority of the study population were young, single males from West African countries, which is representative of the majority of migrants that sought asylum in Italy in 2015 [7, 24]. This pattern is not necessarily representative of all waves of migration to Southern Europe, that tends to be continously evolving. Different groups of migrants come from different parts of Africa, the Middle and Far East at different times. For instance, mainly Syrian and Afghan migrants transited through Greece during 2015 and the start of 2016 [24].

These study’s AS centres hosted almost exclusively men, as many vulnerable people, such as single women, victims of the sex trade, unaccompanied minors, disabled persons, or families were directed to the few dedicated migration centres or bypassed the formal immigration reception system and transited to northern European countries or other places [9], hence were not represented in the study. Women and minors with extreme exploitation stories are a reality as documented by recent humanitarian reports conducted by the International Organisation for Migration and Save the Children [25, 26]. The numbers of young Nigerian women trafficked into prostitution and Egyptian unaccompanied minors are increasing every year [27, 28].

This study shows a high burden of reactive mental health conditions amongst the ASs who were assessed. Post Traumatic Stress Disorder, with related disorders, and depression, were among the most common MH diagnoses. Even though the epidemiological context and demographic population were different, the diagnoses were similar to a cross-sectional study based on a heterogeneous sample drawn from the Swiss national register of asylum seekers and to a descriptive study in a British community mental health service [9, 10]. We saw very few pre-existing MH disorders as problems identified were related to displacement, multiple losses and difficulty in adaptation. All the psychologists who worked in the programme noted a remarkable level of resilience amongst the ASs they assessed [29]. We are aware of the risk of overdiagnosing clinical mental disorders instead of reactive psychological distress. As already highlighted by other authors, emotional distress and psychosocial problems do not imply that the person has a mental disorder, but the large majority of epidemiological surveys of mental disorders and distress have been unsuccessful in accurately distinguishing between the two types [19, 30].

The frequency of potentially traumatic events experienced was also very high and physical stigmata of abuse and violence were evident on the ASs’ bodies [31]. The migration journey was reported as particularly dangerous in terms of risk for own life, detention and exploitation. In this study over half of the ASs had travelled for more than 12 months, which suggests that many had spent most of that time in war-torn Libya where persecution of migrants of sub-Saharan African origin is extremely frequent [8].

There are multiple stressors of adjustment for anyone forced to leave their homeland, but the high levels of potentially traumatic events experienced and accumulated by these migrants before and during their journey put their mental health at risk [32]. A European working group has referred to forced migration itself as a process of grief reactions due to multiple losses, whereby the individual experiences loss of certainty and solid parameters such as a personal, social and cultural structure [33]. These losses may be expressed differently in different cultural and linguistic contexts and can be exacerbated by potential traumas along the journey and once settled [34]. Unfortunately, it was not possible to evaluate the impact of the traumas on these ASs’ mental health conditions given their wide range of backgrounds and experiences, as the subgroups would be too small for comparison.

Post-migratory living stressors were common, which is consistent to what has been shown in an Italian primary health care study [35]. The same study showed that people with post-migratory living difficulties were more likely to have PTSD compared with people without difficulties. Likewise, Swedish and Australian cross-sectional studies showed that individuals with PMLDs were more likely to suffer from MH diagnoses that also included depression and anxiety [2, 36, 37]. Some Dutch studies associated long asylum procedures, lack of work and family issues with significant impacts on anxiety, depressive and somatoform disorders [4, 38].

This was confirmed with anecdotal evidence from the project’s psychologists who stated that the life conditions in most AS centres, with lack of prospects, social disconnection and loss of autonomy, creates a fertile ground for the previous potentially traumatic events to take shape and become symptomatic.

Unfortunately it was difficult to find combined transcultural and psychological expertise in the national health service and psychosocial institutions, where this profile rarely exists. Therefore, it was crucial to collaborate with the ethnopsychiatrist of the Italian non-governmental organisation Medici per i Diritti Umani. In meetings with the local authorities and local MH departments all efforts were invested in raising awareness of this needs and in passing on some transcultural knowledge and expertise.

Beside that, collaboration with UNHCR, IOM and Save the Children was important in complementing our MH activities with the socio-legal information and expertise that eased off distress and uncertainty the ASs lived in.

As already mentioned above, training sessions in basic mental health care were offered and given to non-specialised staff working in the AS centres. A few meetings with the local mental health department and general health authorities took place but didn’t develop into a formal relationship. Individual cases were referred with support of MSF cultural mediators in order to overcome language barriers.

Training of the asylum seekers themselves as outreach workers was never formalised but would definitely be an important strategy to adopt in future similar programmes.

There were several strengths to this study.

This study was based on data collected within routine monitoring by an MSF programme, so it likely reflected the reality on the ground and contributes to the understanding of real-life problems. The four psychologists had all training and experience in ethnopsychiatry and were sensitive to transcultural issues. The cultural mediators had all been through similar migrant journeys and their sensitivity and knowledge of language and customs, including those of Italy, helped build a trusting relationship. The sample size was quite large for ASs with MH conditions and there was a small loss to follow-up of patients once started on treatment. The study adhered to the STROBE guidelines [39].

However, there were some limitations.

The programme was not designed to provide a prevalence rate of MH conditions as we assessed a selective group of people, those who chose to attend the psycho-educational sessions and who subsequently self-referred to the mental health services. We also included those who showed extremes of withdrawn or other abnormal behaviours that raised concern amongst the centre-based staff and the MSF psychologists. We might have missed potential patients that did not attend psycho-educational groups or did not recognise the need for MH care. The fact that our MH service was not integrated into the wider medical programme, could have resulted in missing and underdiagnosing potentially unexplained somatic complaints. Beside that, people with severe, decompensated MH disorders were referred beforehand to the few places available in specialised centres for psychiatric ASs or to a psychiatrist so might bias the study results.

There was lack of standardisation of the screening process, MH diagnoses, case definition for traumatic events and post-migratory living difficulties. At the outset, the psychologists tried to use a screening tool, the validated SRQ-20, but soon abandoned it as it was unsuitable for such a cultural context due to the extreme variability of geographical, cultural, linguistic, social origin of the ASs, despite our attempts to have it translated by the cultural mediators. Although this tools is used worldwide, in our experience the list of questions was often misunderstood and perceived as intrusive by the ASs, causing further suffering and distress reactions, all counterproductive for building up a fruitful therapeutic relationship. Moreover, this questionnaire was conceived for self administration, which limited its utility due to illiteracy among the ASs. In the absence of an alternative standardised screening tool for such a context, the psychologists continued screening for MH symptoms using their clinical judgement.

No structured psychiatric clinical instrument, such as the Structured Clinical Interview (SCID), was used; a semi-structured interview format was adopted instead.

There was also lack of diagnostic standardisation and inter-rating reliability amongst the four psychologists. This was mainly due to operational constraints related to workload, limited time and human resources, as well as geographical spread. Trying to translate ethnic-based conditions into western-based mental health categories (DSM-5) might have resulted in loss of some of their meaning and details, but such a standard reference was required by MSF and the local asylum authorities.

Potentially traumatic events were not systematically explored, but recorded just when spontaneously reported by the patient, likewise, post-migratory living difficulties were recorded only when relevant to the patient’s MH conditions, therefore they might not be complete.

Organizational constraints in such a programme were considerable: besides the clinical work done in parallel, the two clinical psychologists had to be versatile in organising the group and individual MH sessions with specific cultural mediators, travelling several hundreds of kilometres a week across the Sicilian countryside, emotionally debriefing the personnel working at the AS centres and more. Ideally, every patient would benefit from the presence of a cultural mediator during their sessions in order to optimise the transcultural understanding but that wasn’t always feasible due to limited human resources, logistics and time. Moreover, the lack of specific training for this emerging professional category makes its technical competencies quite uneven.

There are a number of programmatic issues raised by the study.

First, the situation of newly-arrived ASs in Italy is not the classical humanitarian emergency but the constant influx of thousands of migrants per week and their reception raises prolonged and repeated humanitarian needs. Many of the humanitarian core principles such as the participation of affected population, building on available resources, a multi-layered support and improvement of the mental wellbeing are valid in such a context and should follow the leading humanitarian guidelines [15, 30, 40, 41].

Second, the important burden of MH conditions and potentially traumatic events suggests that Italian authorities should develop a reception system that treats people with respect of their dignity and supports their resilience. Most migrants have remarkable resilience so public health authorities and MH workers must work on establishing the conditions that promote such resilience. MH care that recognises individuals with diverse geographical, cultural, gender, social, demographical origins, should be integrated with the other levels of the reception system. All people involved in the reception system, from the police officers to the medical personnel should be trained to work and relate with culturally diverse populations. Cultural mediators play a key role in acting as a bridge.

Irrespective from the status of international/humanitarian protection the ASs will receive or will be refused, we should not wait to set up activities that ease their distress and eventually reduce the burden on society.

Third, MH screening should be part of the general health assessment for ASs soon after their arrival. To facilitate this, developing cultural awareness of the degree of trauma ASs might have experienced is important. The role of cultural mediators appears to be crucial to achieve effective screening, the MSF programme could not have functioned without them.

Fourth, there needs to be more coordination and collaboration between the various non-governmental organisations, United Nations agencies and local health authorities to train and empower local MH actors and lay-people.

## Conclusion

This first study on mental health problems in a group of recently-arrived ASs in Sicily showed that mental health conditions and potentially traumatic events were common and important. Despite limitations, it suggests that mental health and psychosocial support should be integrated into European medical services provided for ASs on arrival and while awaiting asylum claims. This would address an important and invisible component of ASs’ health needs, fulfil humanitarian obligations, and reduce the burden of assimilation on receiving countries.

## References

[1] UNHCR. World at War Global Trends 2015 in Review https://s3.amazonaws.com/unhcrsharedmedia/2016/2016-06-20-global-trends/2016-06-14-Global-Trends-2015.pdf. Accessed 30 Sept 2016.

[2] Schweitzer R, Melville F, Steel Z, Lacherez P (2006). Trauma, post-migration living difficulties, and social support as predictors of psychological adjustment in resettled Sudanese refugees. Aust N Z J Psychiatry.

[3] Human Right Watch. Libya: Whipped, Beaten and Hung from Trees. 2014. https://www.hrw.org/news/2014/06/22/libya-whipped-beaten-and-hung-trees. Accessed 30 Sept 2016.

[4] Laban CJ, Gernaat HBPE, Komproe IH, Van IDT, De Jong JTVM (2005). Postmigration living problems and common psychiatric disorders in Iraqi asylum seekers in the Netherlands. J Nerv Ment Dis.

[5] Ministero Dell’Interno. Rapporto sull’ accoglienza di migranti e rifugiati in Italia, 2015. http://www.libertaciviliimmigrazione.interno.it/dipim/export/sites/default/it/assets/pubblicazioni/Rapporto_accoglienza_ps.pdf. Accessed 30 Mar 2016.

[6] Platform for International Cooperation on Undocumented Migrants. Country Report, Italy, 2010. http://files.nowhereland.info/713.pdf. Accessed 30 Sept 2016.

[7] Centro Studi e Ricerche IDOS (2015). Dossier statistico immigrazione 2015.

[8] North Africa Mixed Migration Task Force. The fate of young migrants, asylum-seekers and refugees in Libya today. 2015. http://www.mixedmigrationhub.org/wp-content/uploads/2015/11/Conditions-and-Risks-in-Mixed-Migration-in-North-East-Africa.pdf. Accessed 30 Sept 2016.

[9] Trovato A, Reid A, Takarinda KC, Montaldo C, Decroo T, Owiti P, Bongiorno F, Di Carlo S (2016). Dangerous crossing: demographic and clinical features of rescued sea migrants seen in 2014 at an outpatient clinic at Augusta Harbor, Italy. Confl Heal.

[10] Steel Z, Chey T, Silove D, Marnane C, Bryant RA, van Ommeren M (2009). Association of torture and other potentially traumatic events with mental health outcomes among populations exposed to mass conflict and displacement: a systematic review and meta-analysis. JAMA.

[11] Fazel M, Wheeler J, Danesh J (2005). Prevalence of serious mental disorder in 7000 refugees resettled in western countries: a systematic review. Lancet.

[12] Bogic M, Njoku, Priebe S (2015). Long-term mental health of war-refugees: a systematic literature review. BMC Int Health Hum Rights.

[13] Heeren M, Mueller J, Ehlert U, Schnyder U, Copiery N, Maier T (2012). Mental health of asylum seekers: a cross-sectional study of psychiatric disorders. BMC Psychiatry.

[14] Lindert J, Brahler E, Wittig U, Mielch A, Priebe S (2008). Depression, anxiety and posttraumatic stress disorders in labor migrants, asylum seekers and refugees: a systematic overview. Psychother Psychosom Med Psychol.

[15] World Health Organisation Regional Bureau for Europe (2015). Policy brief on migration and health: mental health care for refugees.

[16] Horyniak D, Melo JS, Farrell RM, Ojeda VD, Strathdee SA (2006). Epidemiology of substance use among forced migrants: a global systematic review. PLoS One.

[17] Weaver H, Roberts B (2010). Drinking and displacement: a systematic review of the influence of forced displacement on harmful alcohol use. Subst Use Misuse.

[18] Schwarz-Nielsen KH, Elklitt A (2009). An evaluation of the mental status of rejected asylum seekers in two Danish asylum centers. Torture.

[19] Hassan H, Ventevogel P, Jefee-Bahloul H, Barkil-Oteo A, Kirmayer LJ (2016). Mental health and psychosocial wellbeing of Syrians affected by armed conflict. Epidemiol Psychiatr Sci.

[20] Gerritsen AAM, Bramsen J, Devillé W, van Willigen LHM, Hovens JE, van der Ploeg HM (2006). Physical and mental health of Afghan, Iranian and Somali asylum seekers and refugees living in the Netherlands. Soc Psychiatry Psychiatr Epidemiol.

[21] Kortmann F, Ten Horn S (1988). Comprehension and motivation in responses to a psychiatric screening instrument. Validity of the SRQ in Ethiopia. Br J Psychiatry.

[22] Rasmussen A, Verkuilen J, Ho E. Posttraumatic Stress Disorder Among Refugees: Measurement Invariance of Harvard Trauma Questionnaire Scores Across Global Regions and Response Patterns. Psychol Assess. 2015;27(4):1160-70. 10.1037/pas0000115 PMC4615261 25894706

[23] International Committee of the Red Cross. Definition of Torture 2014. https://www.icrc.org/eng/resources/documents/faq/torture-icrc-definition-faq-2011-06-24.htm. Accessed 30 Sept 2016.

[24] UNHCR. Refugee/Migrant crisis in Europe situation update 2015 http://data.unhcr.org/mediterranean/regional.php. Accessed 30 Mar 2016

[25] IOM. Migration Trends across the Mediterranean: Connecting the Dots. 2015. http://www.altaiconsulting.com/docs/migration/Altai_Migration_trends_accross_the_Mediterranean_v3.pdf. Accessed: 30 Sep 2016.

[26] Save the Children. Piccoli schiavi invisibili, I minori vittime di tratta e sfruttamento. 2016.

[27] Kelly A and Tondo L. Trafficking of Nigerian women into prostitution in Europe ‘at crisis level. The Guardian. 2016. https://www.theguardian.com/global-development/2016/aug/08/trafficking-of-nigerian-women-into-prostitution-in-europe-at-crisis-level?CMP=Share_iOSApp_Other. Accessed: 30 Sep 2016.

[28] Save the Children. As number of lone children fleeing to Italy soars, new report reveals brutal child trafficking practices. 2016. https://www.savethechildren.net/article/number-lone-children-fleeing-italy-soars-new-report-reveals-brutal-child-trafficking. Accessed: 30 Sep 2016.

[29] Young H. Refugees and Mental Health: These people are stronger than us. The Guardian. 2015. https://www.theguardian.com/global-development-professionals-network/2015/sep/14/refugees-and-mental-health-pyschological-support-msf. Accessed: 30 Sep 2016.

[30] Inter Agency Standing Committee (IASC) (2007). IASC guidelines on mental health and psychosocial support in emergency settings.

[31] Dearden L. Migrants being raped, shot and tortured, on desperate journeys to Europe, doctor reveals. The Independent. 2015. http://www.independent.co.uk/news/world/europe/migrants-being-raped-shot-and-tortured-on-desperate-journeys-to-europe-doctor-reveals-10457130.html. Accessed 30 Sept 2016.

[32] Vostanis P (2014). Meeting the mental health needs of refugees and asylum seekers. Br J Psychiatry.

[33] Carta MG, Bernal M, Hardoy MC, Haro-Abad JM (2005). Migration and mental health in Europe (the state of the mental health in Europe working group: appendix 1). Clin Pract Epidemiol Ment Health.

[34] Bhugra D, Gupta S, Bhui, Craig KT, Dogra N, Ingleby JD, Kirkbride J, Moussaoui D (2011). WPA guidance on mental health and mental health care in migrants. World Psychiatry.

[35] Aragona M, Pucci D, Mazzetti M, Maisano B, Geraci S (2013). Traumatic events, post-migration living difficulties and post-traumatic symptoms in first generation immigrants: a primary care study. Ann Ist Super Sanita.

[36] Silove D, Sinnerbrink I, Field A, Manicavasagar V, Steel Z (1997). Anxiety, depression and PTSD in asylum-seekers: associations with pre-migration trauma and post-migration stressors. Br J Psychiatry.

[37] Momartin S, Steel Z, Coello M, Aroche J, Silove DM, Brooks R (2006). A comparison of the mental health of refugees with temporary versus permanent protection visas. Med J Aust.

[38] Laban CJ, Gernaat HBPE, Komproe IH, Schreuders BA, De Jong JT (2004). Impact of a long asylum procedure on the prevalence of psychiatric disorders in Iraqi asylum seekers in The Netherlands. J Nerv Ment Dis.

[39] Von Elm E, Altman DG, Egger M, Pocock SJ, Gøtzsche C, Vandenbroucke JP (2007). Policy and practice the strengthening the reporting of observational studies in epidemiology (STROBE) statement : guidelines for reporting observational studies. Bull World Health Organ.

[40] World Health Organization and United Nations High Commissioner for Refugees (2015). mhGAP Humanitarian Intervention Guide (mhGAP-HIG): Clinical management of mental, neurological and substance use conditions in humanitarian emergencies.

[41] Mental Health and Psychosocial Support for Refugees, Asylum Seekers and Migrants on the Move in Europe, a multi-agency guidance note. December 2015. http://www.mhpss.net/?get=262/English_mhpss_guidance_note_12_01_2016.pdf. Accessed 30 Sept 2016.

